# A practical technique for subacute hemorrhagic pericarditis, a case report

**DOI:** 10.1186/s13019-021-01499-7

**Published:** 2021-05-01

**Authors:** Long Zhao, Ruofeng Hong, Jianbin Fei, Wenyu Yang

**Affiliations:** Cardiovascular Surgery Department, Hwa Mei Hospital, University of Chinese Academy of Sciences; Ningbo Institute of Life and Health Industry, University of Chinese Academy of Sciences, Room 201, Oriental Venice, Haishu District, Ningbo City, Zhejiang Province China

**Keywords:** Subacute hemorrhagic pericarditis, Viscous effusion, Endoscopic surgery, Case report

## Abstract

**Background:**

We used pericardioscope operation for a patient who suffered from subacute hemorrhagic pericarditis which usually have to had a sternotomy.

**Case presentation:**

A pericardioscope was used in the operation rather than sternotomy on a 66-year-old male who was diagnosed with subacute hemorrhagic pericarditis after PCI(Percutaneous Coronary Intervention). He was discharged 7 days after the operation with an uneventfull postoperative course.

**Conclusions:**

We believe that this technique is a safe procedure without any major complications.

**Supplementary Information:**

The online version contains supplementary material available at 10.1186/s13019-021-01499-7.

## Background

Most of the pericardial effusion was drained by pericardiocentesis, but for those with hemorrhagic effusion, especially with blood clot or subacute hemorrhagic pericarditis, effectively drainage was hard to achieve, sternotomy was unavoidable then. To minimize the trauma, we inserted a thoracoscope into the pericardiac space to remove the blood clot for a patient and got a wonderful outcome.

## Case presentation

A 66-year-old male was admitted to Ningbo Hwa Mei Hospital in July 21, 2020 because of repeated pericardial effusion. He underwent PCI due to Coronary Atherosclerotic Heart Disease in July 3, 2020. He complained pectoralgia for 1 week before hospitalization. Echocardiography showed massive pericardial effusion. Percutaneous pericardiocentesis was performed and about 2400 ml hemorrhagic effusion was drained in the next 6 days. Chest Computed Tomography(CT) and echocardiography on August 7th showed massive pericardial hemorrhage with blood clot. Operation was advised. But the patient claimed explicitly that he would rather die than have a sternotomy because of his concern about the incision infection due to the diabetes and obesity and the disunion of the sternum due to the osteoporosis which caused by renal failure he had suffered for several years. So we performed a minimally invasive surgery.

## Method

The patient was placed on his right side and the surgical approach was through the left pleural space. A pneumothorax was created with partial pulmonary collapse to allow an adequate view of the pericardial surface. A 3-cm incision was made at the left 5th intercostal space, in the midclavicular line. The telescope was passed through the incision. We inspected the pleural space and pericardial surface, identified the left phrenic nerve to avoid damage. A pericardiotomy was performed, some of the bloody effusion was drained, but the blood clot has formed adhesion and was difficult to remove, a partial pericardiectomy of 4*4 cm was done. The pericardial space was then inspected through the telescope.

**Fig. 1:**
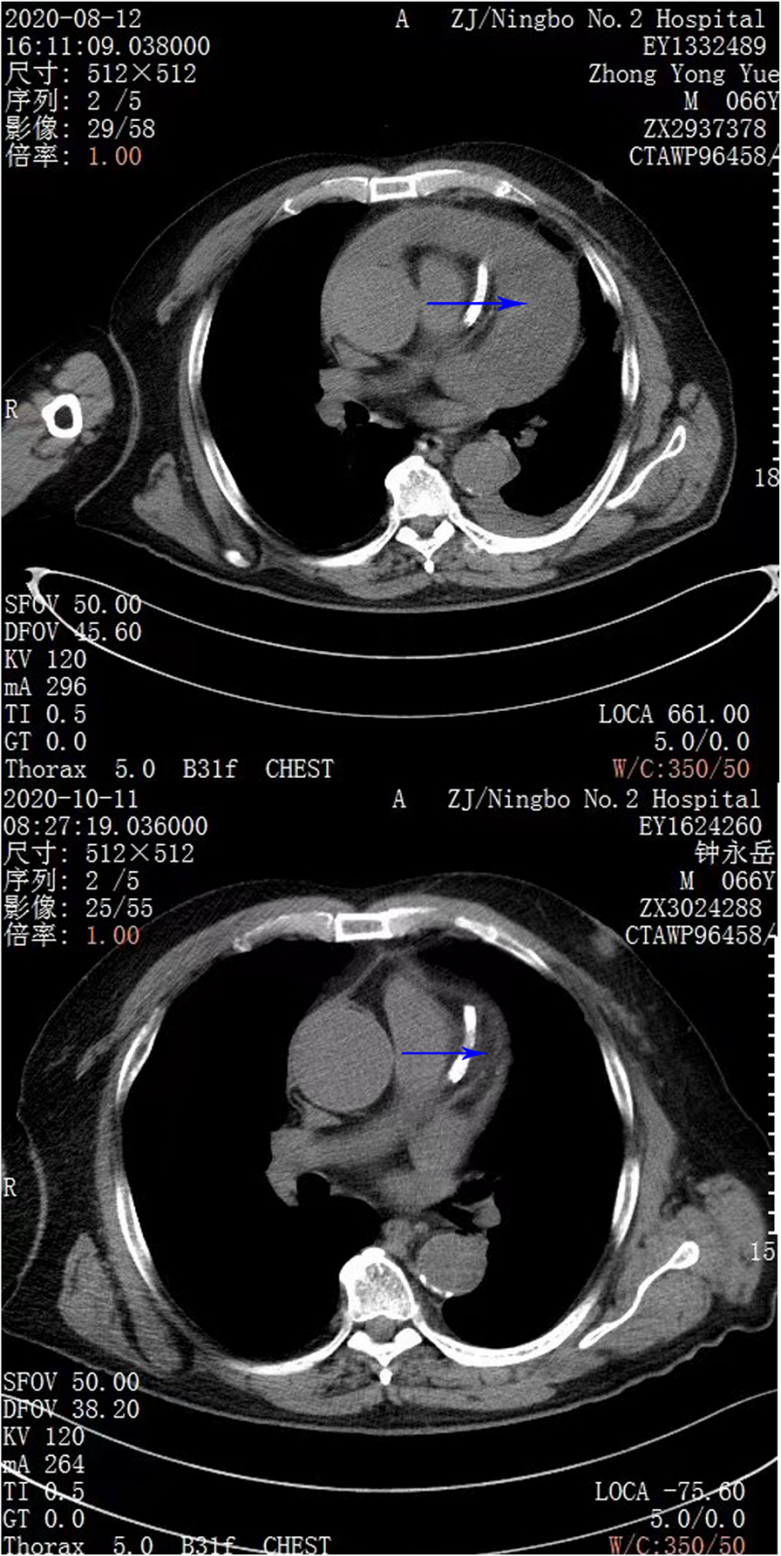
The arrows show the changes before and after the operation, respectively

**Fig. 2:**
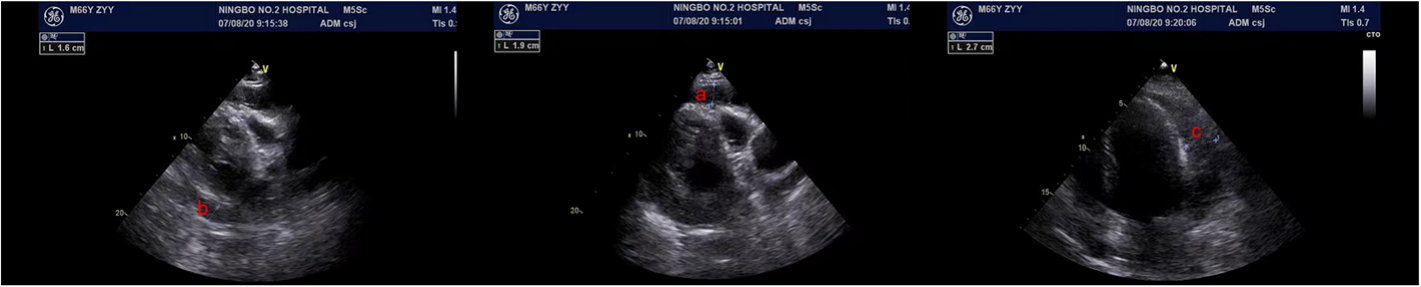
The echocardiography on August 7th shows massive pericardial hemorrhage. a, b and c indicate the thickness of the effusion of the anterior, posterior and left lateral pericardium cavity was 19 mm, 17 mm, and 27 mm, respectively

The pericardium was caught by double joint oval forceps and lift up. After the blood clot under the incision was excavated by another double joint oval forceps, the thoracoscope was inserted into the pericardial cavity through the pericardial incision. Take the advantage of the 30°slope of the thoracoscope, we can observe different directions by turn the scope without move the shaft. To obtain a better view, we used a curved double joint forceps to enlarge the pericardial cavity by using the turning of the forceps to lift the pericardium. After cleaning the left part of the pericardial cavity, we turn the scope to the anterior and right lateral cavity to clean the blood clot until the level of superior vena cava. It is hard to avoid squeezing the heart during the procedure, so the ECG(Electrocardiogram) and blood pressure must be payed attention to during the operation. A drainage tube was insert into the pericardial cavity and the incision was sutured in layers. Another drainage tube was placed in the pleural space under direct vision and connected to underwater seal. The postoperative course was uneventfull. He was discharged 7 days after the operation. A chest CT on October 11th showed there was almost no pericardial effusion left (Fig. 1, Fig. 2).

## Discussion

Pericardial effusion limits cardiac expansion and increases atrial, ventricular, and pericardial pressures. The result is a reduction in cardiac output, and vasoconstriction to maintain the blood pressure [1–3]. A common way to drain the effusion is pericardiocentesis subxiphiodly. However, it is often unable to provide sustainable results with a high rate of recurrence and low rate of diagnosis [4]. The best method to treat the pericardial effusions remains a controversial subject. Nevertheless, with the advancement of minimally invasive procedures in cardiovascular surgery, it has became the first choice and increasing reports have shown the efficacy, safety and reproducibility of the thoracoscopic procedure [5–8]. But for those with viscous effusion, like subacute hemorrhagic pericarditis, the efficiency of pericardial fenestration through a video-thoracoscopic or subxiphoid approach [9] is limited. By inserting the thoracoscope into the pericardial cavity, lifting the parietal pericardium with a double joint forceps can create a promising space to observe and scrape the viscous effusion. Benefit from the 30 degree slope of the lenses of the thoracoscope, a larger vision can be obtained by rotation and propulsion of the shaft. During the procedure, an extra attention must be payed to avoid squeezing the free wall of ventricles roughly so as not to cause ventricular rupture or hemodynamic instability. Other than transient arrhythmias, the procedure is not associated with major complications.

## Conclusion

Pericardioscope is an effective and practical technique for subacute hemorrhagic pericarditis which should be applied in more patients.

## Supplementary Information

**Additional file 1.**

## Data Availability

There is no data in this manuscript.

## Abbreviations

PCI: Percutaneous Coronary Intervention
CT: Computed Tomography
ECG: Electrocardiogram
